# Case Report: Acute Dyspnea in a Young Female

**DOI:** 10.5070/M5.52254

**Published:** 2026-04-30

**Authors:** Brian Kenny, Marwa Ali

**Affiliations:** * Rutgers University New Jersey Medical School, Department of Emergency Medicine, Newark, NJ

## Abstract

Sarcoidosis is a multisystem inflammatory disease characterized by non-caseating granulomas, primarily affecting the lungs but also involving other organs such as the skin, eyes, and heart.^1^ Due to its variable presentation, diagnosing sarcoidosis in the emergency department (ED) can be challenging. We report a case of a 28-year-old African American female presenting with acute dyspnea on exertion, significant weight loss, and multiple cutaneous nodules. Her diagnostic workup revealed hypercalcemia, diffuse pulmonary nodules, and extensive lymphadenopathy on imaging. Biopsy of her eyelid lesions confirmed non-caseating granulomas, leading to a diagnosis of sarcoidosis. She was initiated on high-dose steroids and evaluated for further immunologic therapy. Sarcoidosis presents a diagnostic challenge due to its nonspecific symptoms, often mimicking more common conditions.^2^ Early recognition in the ED is critical to prevent disease progression. This case underscores the importance of considering sarcoidosis in patients with unexplained systemic symptoms and highlights the need for a multidisciplinary approach to management.

**Topics:**

Sarcoidosis, respiratory distress, emergency medicine.

**Figure:**
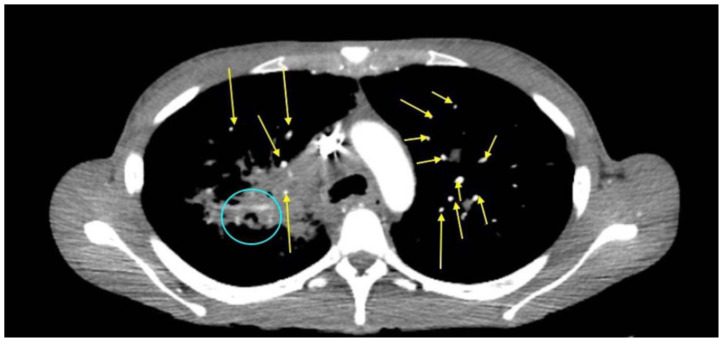


**Figure:**
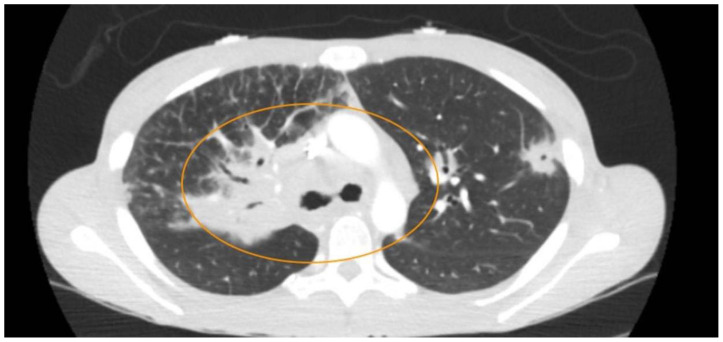


## Brief introduction

Sarcoidosis is a multisystem inflammatory disease marked by the formation of non-caseating granulomas in affected tissues. The exact cause remains unknown; however, it is believed to stem from an exaggerated immune response to environmental or infectious triggers in a genetically predisposed individual.^1^,^2^ The primary organs involved are the lungs, but the disease can also impact the skin, eyes, heart, nervous system, and other organs.^3^,^4^

In the ED, diagnosing sarcoidosis is challenging due to the highly variable presentation in the patient. Most cases that remain asymptomatic are incidentally discovered, whereas others may be discovered in patients with acute respiratory symptoms or an organ-specific dysfunction. Given that sarcoidosis has similar symptoms to more common emergency conditions, there must be a high index of suspicion, particularly in patients with unexplained dyspnea, cough, or systemic inflammatory signs.^2^ With a worldwide distribution, this condition occurs most frequently among females aged 20 to 40 years; in the United States, African Americans are disproportionately affected, with an incidence of approximately 35 new cases per 100,000 individuals.^5^ Sarcoidosis is often diagnosed only after multiple visits to the ED or a primary care physician due to its rarity (approximately 140 cases per 100,000 individuals worldwide).^5^

This case report explores the clinical manifestations of a young female who presented to the ED with dyspnea on exertion and multiple skin lesions. Recognizing sarcoidosis early in the emergency setting can facilitate timely referral to specialists and prevent disease progression and complications.

## Presenting concerns and clinical findings

We present a 28-year-old, African American female who presented to the ED with a six-month history of weight loss and acute dyspnea on exertion. The patient reported that she has had decreased appetite which has resulted in a loss of 110 pounds in the preceding six months. Additionally, the patient stated that she was unable to walk up the stairs in her apartment today, which prompted the ED visit. Over the past two months, the patient has noticed that she has been developing painful nodules throughout her body, mainly located on the bilateral arms, legs, and eyelids.

The patient presented tachycardic with a heart rate in the 120s, normotensive, oxygen saturation of 95% on room air, and afebrile. She was in no significant distress; she was noted to have clear lungs bilaterally, no abdominal tenderness, and no focal neurological deficits. On skin exam, there were multiple 1–2 cm painless, non-mobile nodules on her arms, legs, and eyelids. The nodules were skin-colored, without surrounding color change or signs of acute infection.

## Significant findings

Her diagnostic workup included a combination of both laboratory and diagnostic imaging. Her labs were significant for no leukocytosis, hemoglobin within normal limits, calcium of 10.7 mg/dl (Ref: 8.4–10.2 mg/dl), Parathyroid Hormone 6.7 pg/ml (Ref: 8.7–77.1 pg/ml). Additionally, the patient’s HIV, rapid plasma reagin test (RPR), and pregnancy tests were negative. The patient underwent computer tomography (CT) imaging of the chest, abdomen, and pelvis. Computer tomography of the chest showed bilateral diffuse pulmonary nodules in a perilymphatic distribution (yellow arrows), and diffusely increased mediastinal and hilar soft tissue densities (blue circle), likely representing lymphadenopathy; a left upper lobe lesion with central cavitation (orange circle) was also seen likely to be associated with the same disease process as the extensive lymphadenopathy. Imaging of the abdomen and pelvis was significant for diffuse hypodensities of the liver and spleen suggesting a multi-system process.

## Patient course

The patient was subsequently admitted to the hospital with both pulmonary and ophthalmologic consultations. The patient was started on high-dose steroids and had her work-up broadened to prepare for immunologic medication administration. Given that sarcoidosis is a diagnosis of exclusion, the patient had an extensive workup and skin lesion biopsy for further objective information to suggest sarcoidosis. The patient had bilateral eyelid biopsies from ophthalmology showing non-caseating granulomas. The patient was treated with intravenous fluids for hypercalcemia. The patient was hospitalized for three days and received inpatient stabilization consisting of intravenous corticosteroids and was discharged with pulmonology and ophthalmology follow up.

## Discussion

Sarcoidosis is a systemic inflammatory disease that can affect multiple organ systems. Patients often present with a variety of complaints, and these symptoms can mimic more common emergency conditions, leading to delays in the diagnosis of sarcoidosis.^1^ The primary organs affected are the lungs, but patients frequently exhibit skin lesions characteristic of erythema nodosum, lupus pernio, or various plaques and papules across the body.^1^,^2^ Because sarcoidosis often presents with nonspecific symptoms—such as shortness of breath, fatigue, rash, and lymphadenopathy—that overlap with many common emergency department chief complaints, clinicians must maintain heightened clinical awareness to recognize and diagnose this condition in the ED. Patients are frequently well-appearing; therefore, once emergency conditions are ruled out, they are safely discharged from the ED, but they often return due to the progressive and chronic nature of the disease.

Sarcoidosis is an inflammatory disorder characterized by multi-organ involvement, with the hypothesized role of human leukocyte antigen (HLA) groups in genetics and environmental exposures as contributing factors. The disease is thought to begin in the lungs, where the activation of macrophages results in the downstream recruitment of cluster differentiation 4 (CD4) T cells that develop non-caseating granulomas.^3^,^4^ The chief complaints are broad and variable, and they should be considered by the emergency department physician in this subgroup of patients. Twenty-five percent of patients will present with neurosarcoidosis, manifesting as psychiatric disturbances, altered consciousness, or seizures due to granulomatous infiltration into the central nervous system.^6^,^7^ Up to 83% of patients will have eye complaints, ranging from lacrimal duct infiltration to uveitis or optic neuritis, with each condition treated symptomatically.^8^ Only about 5% of patients will have cardiac involvement, which presents variably with conduction abnormalities, myocarditis (fever, malaise, chest pain), pericardial effusion, or acute valvular abnormalities.^9^–^12^ One-quarter of patients will present with cutaneous lesions, most commonly in the form of erythema nodosum or lupus pernio.^13^ Seventy percent of patients will experience salivary gland involvement, resulting in painless enlargement of the parotid glands.^14^,^15^ Additionally, these patients may exhibit an array of electrolyte abnormalities secondary to infiltration of the thyroid, parathyroid, and adrenal glands.^16^

Given the highly variable presentation, often not present all at the same time, patients are ruled out for an emergent condition and instructed to seek follow-up care. Often the patients are treated with symptomatic care and will be admitted for further diagnostic testing prior to starting immunological therapy since sarcoidosis is a diagnosis of exclusion and results in chronic immunosuppressant treatment. Without recognition and treatment of the disease, it will continue to progress, ultimately leading to death if not identified. It is imperative for an emergency physician to recognize multisystem involvement (i.e., pulmonary, dermatologic, ocular, cardiac and neurological) in order to diagnose sarcoidosis in the ED.^6^–^16^ Additionally, reviewing chest imaging for unexplained hilar and/or mediastinal lymphadenopathy without a clear infectious source should raise the suspicion for sarcoidosis, especially in higher-risk populations.^4^ It is important to keep this disease on the differential in patients presenting with a constellation of symptoms and involve consultants early for proper treatment.

## Supplementary Information






