# Supplementary figures and images for: Case Report: Acute Dyspnea in a Young Female
Source: J Educ Teach Emerg Med. 2026 Apr 30;11(2):V6–9. doi: 10.5070/M5.52254 (PMC13152371; doi:10.5070/M5.52254)

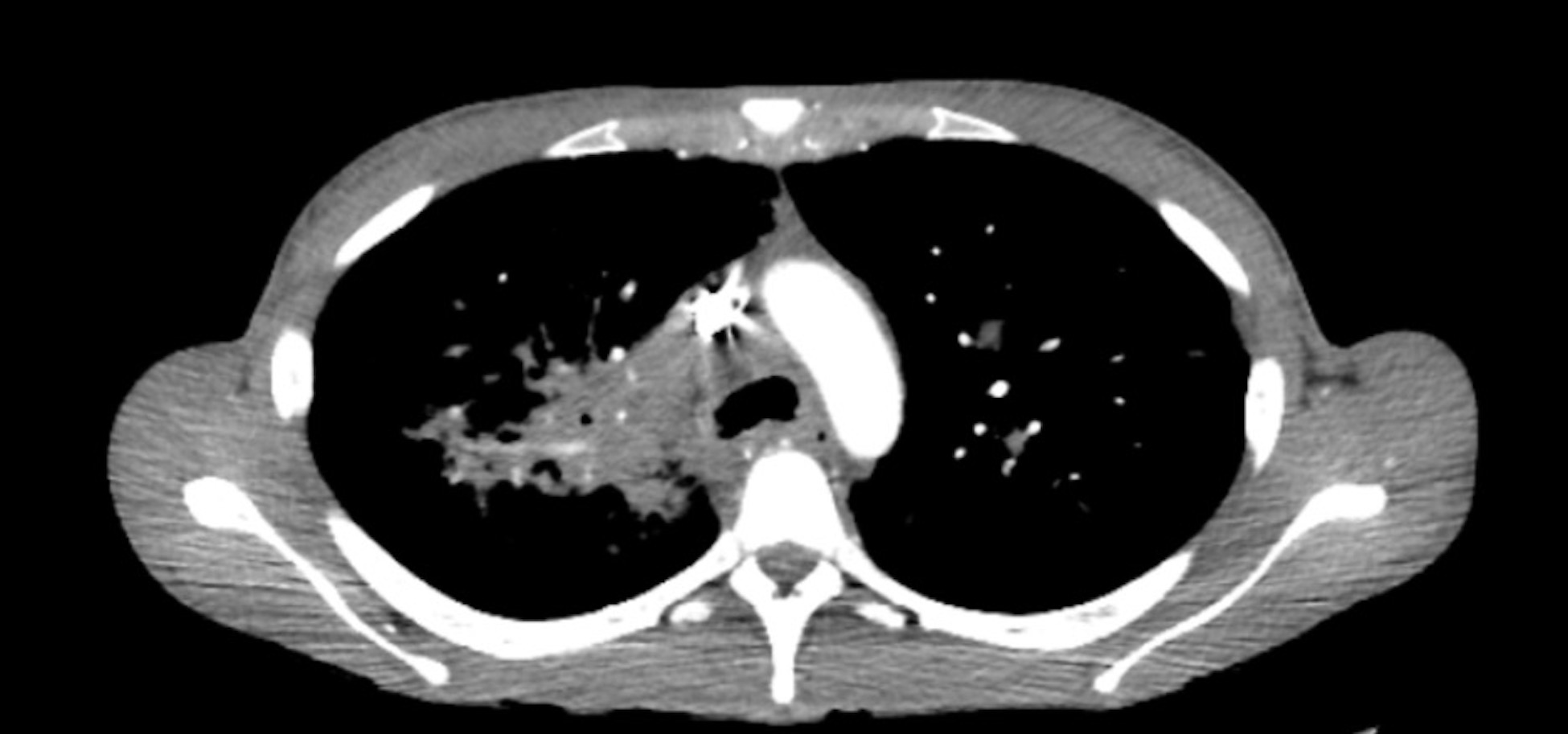

Supplement: Supplementary file 1 [file 11-2-V6-Supp1.jpg]

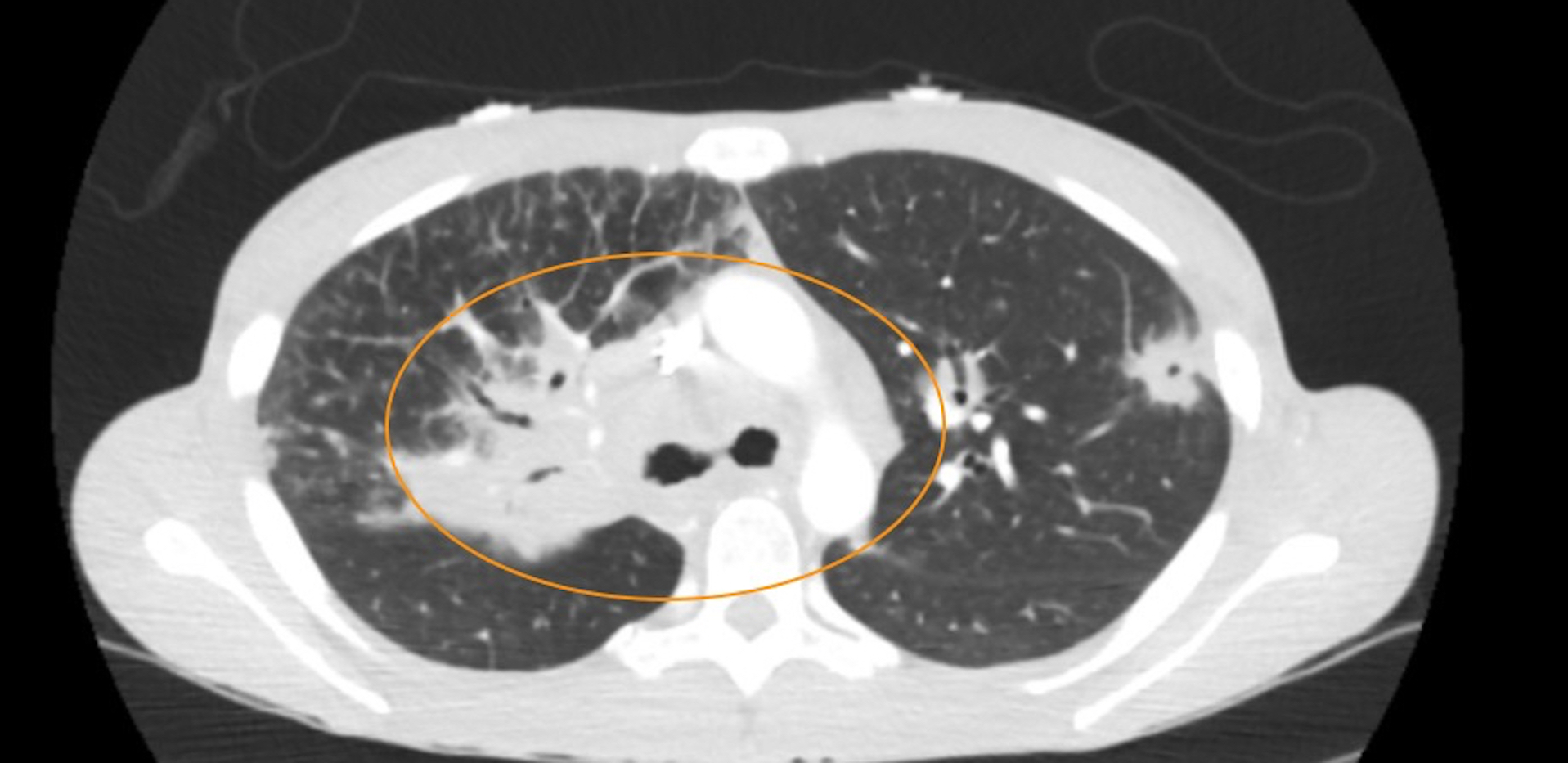

Supplement: Supplementary file 2 [file 11-2-V6-Supp2.jpg]

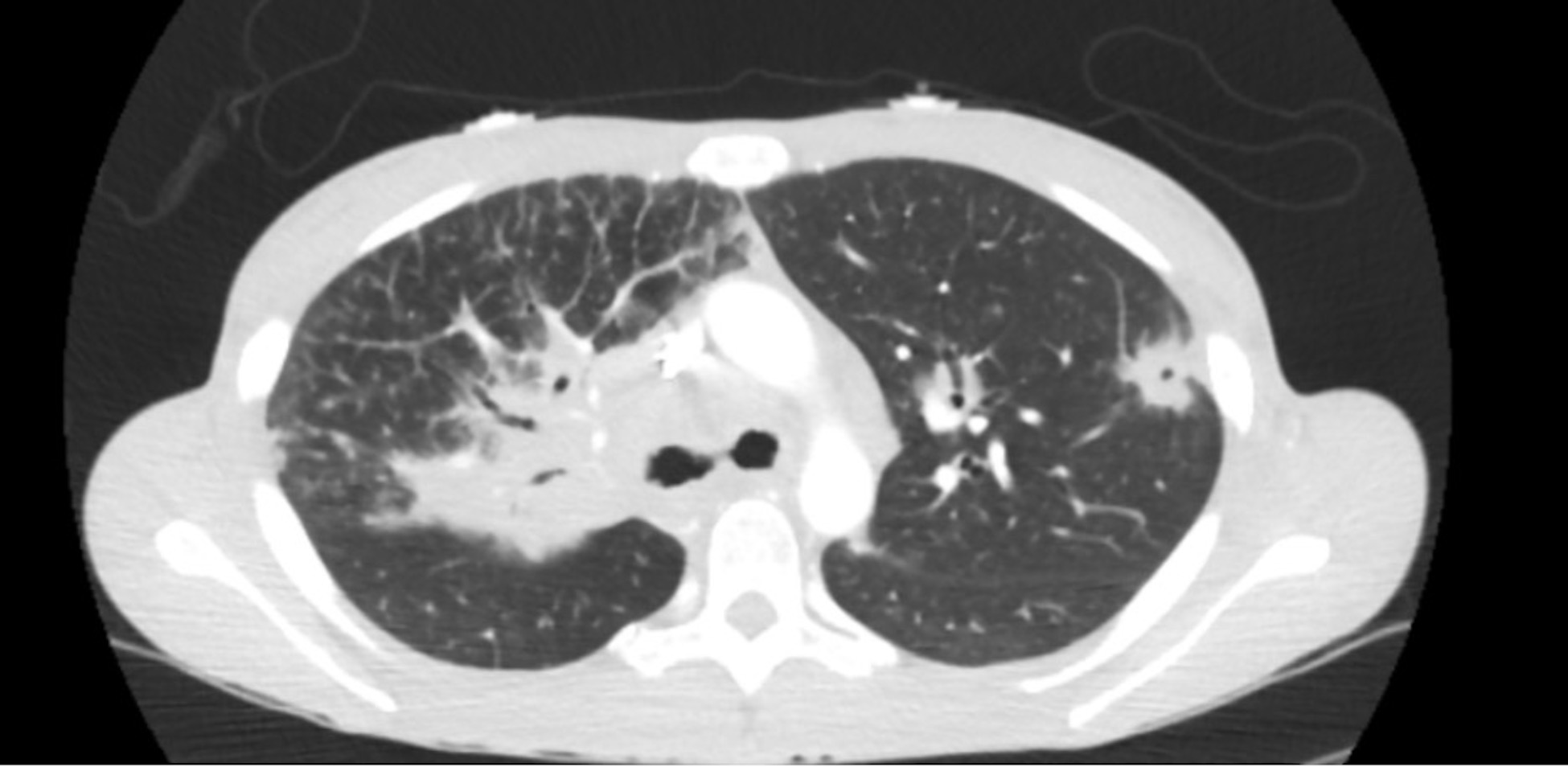

Supplement: Supplementary file 3 [file 11-2-V6-Supp3.jpg]
